# Corticospinal and peripheral responses to heat-induced hypo-hydration: potential physiological mechanisms and implications for neuromuscular function

**DOI:** 10.1007/s00421-022-04937-z

**Published:** 2022-04-01

**Authors:** Nasir Uddin, Jamie Tallent, Stephen D. Patterson, Stuart Goodall, Mark Waldron

**Affiliations:** 1 Centre for Applied Performance Sciences, Faculty of Sport, Health and Applied Sciences, St Mary’s University, Twickenham, UK; 2 School of Human Sciences, London Metropolitan University, London, UK; 3 School of Sport, Rehabilitation, and Exercise Sciences, University of Essex, Colchester, UK; 4 Department of Physiotherapy, Faculty of Medicine, Nursing and Health Sciences, School of Primary and Allied Health Care, Monash University, Clayton, Australia; 5 Department of Sport, Exercise, and Rehabilitation, Northumbria University, London, UK; 6 Applied Sports Science Technology and Medicine Research Centre (A-STEM), College of Engineering, Swansea University, A120 Engineering East, Bay Campus, Swansea, SA1 8EN Wales UK; 7 School of Health and Behavioural Sciences, University of the Sunshine Coast, Sippy Downs, QLD Australia; 8 Welsh Institute of Performance Science, Swansea University, Swansea, UK

**Keywords:** Dehydration, Electromyography, Fatigue, Hyperthermia, Transcranial magnetic stimulation

## Abstract

Heat-induced hypo-hydration (hyperosmotic hypovolemia) can reduce prolonged skeletal muscle performance; however, the mechanisms are less well understood and the reported effects on all aspects of neuromuscular function and brief maximal contractions are inconsistent. Historically, a 4–6% reduction of body mass has not been considered to impair muscle function in humans, as determined by muscle torque, membrane excitability and peak power production. With the development of magnetic resonance imaging and neurophysiological techniques, such as electromyography, peripheral nerve, and transcranial magnetic stimulation (TMS), the integrity of the brain-to-muscle pathway can be further investigated. The findings of this review demonstrate that heat-induced hypo-hydration impairs neuromuscular function, particularly during repeated and sustained contractions. Additionally, the mechanisms are separate to those of hyperthermia-induced fatigue and are likely a result of modulations to corticospinal inhibition, increased fibre conduction velocity, pain perception and impaired contractile function. This review also sheds light on the view that hypo-hydration has ‘no effect’ on neuromuscular function during brief maximal voluntary contractions. It is hypothesised that irrespective of unchanged force, compensatory reductions in cortical inhibition are likely to occur, in the attempt of achieving adequate force production. Studies using single-pulse TMS have shown that hypo-hydration can reduce maximal isometric and eccentric force, despite a reduction in cortical inhibition, but the cause of this is currently unclear. Future work should investigate the intracortical inhibitory and excitatory pathways within the brain, to elucidate the role of the central nervous system in force output, following heat-induced hypo-hydration.

## Introduction

With growing concerns of occupational heat-stress, increased recreational and sports participation in hotter climates, there is increasing interest in the challenges posed by high ambient temperatures and the ensuing threat of hypo-hydration on aspects of physical function, such as that of the neuromuscular system. Indeed, water plays a crucial role in cellular homeostasis, with transient loss of dissolved substances in body fluid leading to alterations in osmolality and, consequently, water distribution across neural and skeletal muscle cell membranes. Increases in body temperature, incurred due to exercise-induced metabolic heat gain, or high ambient temperatures, triggers a thermo-effector sweating response (Romanovsky 2007). Typical thermoregulatory sweating, coupled with inadequate fluid intake, can result in hypotonic fluid losses from extracellular fluid in relation to blood plasma, leading to an osmotic gradient, thus facilitating transmembrane flow of fluid from the intracellular fluid space towards the extracellular fluid space (Costill et al. 1976; Durkot et al. 1986). This process of fluid loss in intracellular fluid (and the hypertonic characteristics of the extracellular fluid) is referred to as hypertonic hypovolemia or intracellular dehydration (Adolph et al. 1947; Lee and Mulder 1935; Pearcy et al. 1956) and has likely implications on neuromuscular function.

In addition to autonomic feedback loops regulating bodily fluid balance (Andreoli et al. 2010), it is thought that several complex regulatory mechanisms protect neuronal tissue from transient fluid-shifts. However, recent studies employing functional magnetic resonance imaging (fMRI) have demonstrated transient brain anatomical alterations, consistent with fluid loss (Kempton et al. 2009, 2011; Streitburger et al. 2012) and increased neuronal activation to achieve a similar cognitive output (when euhydrated) (Kempton et al. 2011). Furthermore, hypo-hydration results in a reduction of maximal isometric force (Bowtell et al. 2013; Ross et al. 2012), time to exhaustion during repeated submaximal contractions (Montain et al. 1998; Bigard et al. 2001; Barley et al. 2018), and reductions in endurance performance (El Helou et al. 2012; James et al. 2017; Adams et al. 2018; Funnell et al. 2019; Campa et al. 2020). Interestingly, force decrements are observed despite reported increases in muscle excitability, unchanged corticospinal excitability (Bowtell et al. 2013), unchanged voluntary activation (Del Coso et al. 2008; Periard et al. 2012; Barley et al. 2018) or increased central activation (Bigard et al. 2001). Though hypo-hydration notably reduces exercise performance via increased cardiovascular strain (González-Alonso et al. 1997), reduced blood flow, aerobic metabolism (Cheuvront et al. 2010) and thermoregulatory function (Casa 1999), the neuromuscular responses to hypo-hydration are less well understood—in part, due to the combined effects of hyperthermia—and speculated to be a result of ionic imbalances (Sjoogard et al. 1985; Casa 1999), reduced muscle contractility and increased central fatigue (Bigard et al. 2001). Whilst some work has reported electromyographical (EMG) responses to hypo-hydration, the corticospinal, supraspinal, and morphological changes (in the central nervous system [CNS]) observed following heat-induced hypo-hydration have received less attention. With the increased specificity of neurophysiological techniques, such as transcranial magnetic stimulation (TMS) and motor nerve stimulation (MNS), the current review aims to summarise these findings and shed light on the integrity of the brain-to-muscle pathway following heat-induced hypo-hydration (with and without the effects of hyperthermia). Furthermore, we propose the various sites and mechanisms of neuromuscular impairment following intracellular dehydration, and briefly discuss the methodological limitations and scope for future studies.

## Central and peripheral responses to hypo-hydration

### Brain and spinal cord-specific responses

In contrast to the intracellular fluid losses observed in most mammalian tissue (i.e., muscle, skin, gut) during dehydration, early research conducted in animal models reported that severe hypo-hydration (10–15% total body weight) and hyperosmolality, elicited minimal (Hamilton and Schwartz 1935; Wallace et al. 1970) or no reductions in brain water content (Nose et al. 1983; Arieff et al. 1977). However, recent research investigating the effects of hydration status on brain and spinal cord tissue have observed transient anatomical alterations in moderately hypo-hydrated humans (Duning et al. 2005; Nakamura et al. 2014; Wittbrodt et al. 2018; Streitburger et al. 2012; Kempton et al. 2009, 2011; Dickson et al. 2005; Biller et al. 2015; Wang et al. 2014; Tan et al. 2019). Hypo-hydration is consistent with reductions in spinal cord cross-sectional area (Wang et al. 2014), brain volume (Duning et al. 2005; Nakamura et al. 2014; Wittbrodt et al. 2018; Streitburger et al. 2012), and brain ventricular expansion (proportionate to body mass loss; Kempton et al. 2009, 2011; Dickson et al. 2005), indicating in vivo fluid losses from brain and spinal cord tissue. Therefore, heat-induced hypo-hydration leads to a reduction in brain and spinal cord volume and ventricular expansion, resulting in acute anatomical alterations. In addition, the increase in PaCO_2_ secondary to heat-induced hypo-hydration may lead to reductions in cerebral blood volume and flow during exercise (Trangmar et al. 2014, 2015), which in turn, increases oxygen extraction, suggesting a heightened cognitive effort to maintain physiological output (Trangmar and Gonzalez-Alonso 2017, 2019). Indeed Kempton et al. (2011) demonstrated hypo-hydration resulted in increased ventricular volume and neuronal activity in the fronto-parietal region (using blood-oxygen-dependent-level functional magnetic resonance imaging [fMRI] signal), during a cognitive task; however, the effect of acute anatomical alterations and reduced cerebral blood flow on neuromuscular function remains unknown. Furthermore, given the poor temporal resolution of MRI for rapid movement (Asakawa et al. 2003), it is possible that alternative techniques are required to measure rapid muscle contractions.

Brain activation (in the context of skeletal muscle function) can be further investigated by measures of corticospinal excitability (CSE), utilising TMS; however, little is known of corticomotor activity (elicited through TMS) after hypo-hydration. CSE is determined using the EMG-derived amplitude of a motor evoked potential (MEP), and when normalised to the compound muscle action potential (*M*_max_; using MNS) represents the summed excitability along the brain-to-muscle pathway (MacKinnon and Rothwell 2000; Pascual-Leone et al. 1995). The corticospinal silent period (cSP), elicited during contraction, is also an EMG-derived measurement of inhibition, referring to an interruption of voluntary EMG in the presence of a muscle contraction, and is most likely related to increased corticospinal inhibition, mediated by inhibitory γ-aminobutyric acid (GABA_B_) receptors (Wolters et al. 2008; Yacyshyn et al. 2016). In addition, voluntary activation (VA) can be assessed by superimposing TMS (VA_TMS_) on a maximal voluntary contraction (MVC), thus when TMS evokes an increase in force production, it signifies a suboptimal output from the motor cortex to maximally activate the motoneurone pool (i.e., supraspinal fatigue; Gandevia 2001). Bowtell et al. (2013) investigated the effects of hypo-hydration and euhydration (after exercise in the heat) on corticomotor output. No changes were observed in VA_TMS_, CSE and cSP among hypo-hydrated subjects despite a reduction in force; however, the cSP was lengthened in euhydrated subjects, indicating reduced corticospinal inhibition after hypo-hydration. Collectively, it can be suggested that hypo-hydration does not elicit any changes to motor cortical output but could reduce cortical inhibition during active muscle contractions; however, the reasons for this are unclear.

### Muscle contractility-specific responses

As with the brain and spinal cord, morphological alterations are observed in skeletal muscle (reduced cross-sectional area and overall volume) during hypo-hydration (Nose et al. 1983; Hackney et al. 2012; Farhat et al. 2018) which could explain a reduction in maximum force production (Ikai and Fukunaga 1968; Knuttgen 1976). Muscle contraction time and half-relaxation time (HRT) reflect the rate of cross-bridge cycling and the release/uptake of calcium ions (Ca^2+^) from the sarcoplasmic reticulum (SR), respectively (Close 1972). In rats, 96 h of water deprivation led to increased tetanic tension relative to euhydrated rats, with no change in muscle contraction time and HRT, despite a 10% reduction of the soleus mass, indicating a compensatory pathway to preserve neuromuscular function (Farhat et al. 2018). Additionally, VA measured with motor nerve stimulation (VA_MNS_) can elicit extra force during an MVC, when voluntary drive of α-motoneurones is inadequate. VA_MNS_ is notably unaffected by hypo-hydration (2–5% body mass) (Barley et al. 2018; Bowtell et al. 2013; Periard 2012; Stewart et al. 2014), therefore it is unlikely that fluid losses lead to a reduction in spinal motor neuron discharge (i.e., spinal fatigue). Minshull and James (2013), reported a ~ 8% reduction in maximal voluntary contraction (MVC) force following 24-h fluid restriction, yet no changes in evoked force, rate of force development, and electromechanical delay, indicating minimal changes to the excitation–contraction coupling (ECC) process. Interestingly, data are varied in humans, with reports of no changes (Greiwe et al. 1998; Montain et al. 1998; Evetovich et al. 2002; Barley et al. 2018; Periard et al. 2012) or reductions in peak strength and voluntary force production in response to heat-induced hypo-hydration (Bosco et al. 1968; Torranin et al. 1979; Webster et al. 1990; Judelsen et al. 2007; Hayes and Morse 2010; Schofstall et al. 2001; Bigard et al. 2001; Bowtell et al. 2013). Bowtell et al. (2013) reported an increase in sarcolemma excitability (M-Wave amplitude) during MVCs, yet there was a reduction in muscle torque and increased HRT. This indicates a disruption to the ECC process and efficiency of the release and reuptake of Ca^2+^ from the SR, irrespective of neural drive and a compensatory increase in muscle membrane excitability. A plausible mechanism for why muscle force is reduced, despite increased sarcolemma excitability, has not been proposed. However, this suggests that force production, despite increased neural drive and sarcolemma excitability after hypo-hydration, may be impaired at a contractile level.

### Distinguishing between specific responses of hyperthermia and hypo-hydration

A methodological limitation of inducing intracellular dehydration is the use of heat stress and exercise, resulting in the possible effects of hypo-hydration being masked or exacerbated by that of hyperthermia and exercise-induced fatigue (Judelsen et al. 2007). This section will summarise the independent effects of hyperthermia and hypo-hydration on measures of neuromuscular function.

Cerebral neuronal activity can be ascertained from electroencephalography (EEG), which is notably distinguished from neural imaging techniques, such as MRI, due to superior resolutions in temporal neural networks (Crosson et al. 2010). In clinical practice, cerebral activity obtained from EEG is subdivided into several bandwidths to signify the location of the acquired signal and brain state. Beta waves are predominantly located in the frontal region and represent a state of alertness and focus, whilst alpha waves are associated with relaxation and inhibition (Tatum 2007). Several studies have investigated the effects of hyperthermia with dehydration and exercise (Ftaiti et al. 2010) and without dehydration (Nielsen et al. 2001; Nybo and Nielsen 2001) on EEG activity, reporting an increased alpha and decreased beta power during prolonged exercise, potentially indicating increased inhibitory activity in pyramidal neurons. This agrees with van den Heuvel et al. (2020), who investigated EEG changes after passive hyperthermia with and without dehydration, and found no independent effect of hypo-hydration on resting EEG, suggesting neural alterations to be related to thermoregulatory factors. In addition, Caputa et al (1986) reported heightened hypothalamic temperatures (42–43 °C) led to a reduction in exercise capacity in animals; however, trunk temperatures (below 43.5 °C) were unrelated to exercise capacity, indicating a failure of central origin during hyperthermia. These data may partially explain the observations of supraspinal fatigue, after exercise in the heat (Goodall et al. 2015; Todd et al. 2005; Ross et al. 2012; Periard et al. 2014a, b). Collectively, passive and exercise-induced hyperthermia results in increased inhibitory brain activity during rest and prolonged exhaustive exercise. However, this is independent of hypo-hydration and might not reflect brain activity during brief and sustained MVCs. Further studies are required to elucidate brain activity during brief and sustained bouts of maximal strength, and to establish if there are differing mechanisms of hypo-hydration and hyperthermia which lead to force decrements.

Studies in which hyperthermia is induced either passively (Morrison et al. 2004; Racinais et al. 2008; Saboisky et al. 2003; Todd et al. 2005) or actively (Del Coso et al. 2008; Periard et al. 2011, 2014a, b; Goodall et al. 2015) without hypo-hydration, suggest a significant contribution of spinal and peripheral components to fatigue. Passive or active hyperthermia result in a reduction of MVCs, which is accompanied by reduced VA, H-reflex and M-wave amplitudes implicating altered supraspinal, spinal and peripheral excitatory output, respectively (for review, see Racinais and Oksa 2010). Therefore, it is likely that a reduction of VA is attributed to hyperthermia only; as evidenced by Morrison et al. (2004), who demonstrated the restoration of VA to baseline values after cooling. In addition, despite a reduction of VA after hyperthermia and hypo-hydration, fluid restoration had no effect on VA (Del Coso et al. 2008). This is in agreement with various hypo-hydration studies (Periard et al. 2012; Bowtell et al. 2013; Barley et al. 2018) and suggests VA is unaffected by hypo-hydration (2–5% body weight). Interestingly, hyperthermia also leads to an increased muscle relaxation rate and decreased muscle half-relaxation time (Todd et al. 2005; Periard et al. 2014a, b), yet it is reported that a centrally mediated rate of activation is sufficient to overcome the faster relaxation rate (Periard et al. 2014a, b). Conversely, hypo-hydration leads to an unchanged half-relaxation time (Barley et al. 2018), muscle relaxation rate or increased half-relaxation time (Bowtell et al. 2013). In addition, Bowtell et al (2013) reported an increased M-wave amplitude and reduced corticospinal inhibition (relative to euhydrated participants) during an MVC after hypo-hydration, yet a deficit in muscle torque persisted, indicating an inadequate voluntary drive to activate sarcolemmal action potentials and the cross-bridge cycle as a potential site of contractile failure. While the reports of reduced maximal strength are varied, it is important to note that this is the result of a mixed body of work examining exercise performance, alongside factors which might mask, or exacerbate, the effects of hypo-hydration (e.g., ambient temperatures and caloric restriction) (Judelsen et al. 2007). When accounting for these factors, Judelsen et al. (2007) concluded that hypo-hydration caused a 2 and 3% reduction in strength and power, respectively. These findings indicate distinctive mechanisms (related to contraction failure) when intracellular water has not been restored, which may differ from neural and contractile alterations during hyperthermia.

The next section summarises some of the proposed physiological mechanisms that explain the modulation of intracortical circuitry and reduction of force after heat-induced hypo-hydration.

## Potential physiological mechanisms

### Disrupted fibre conduction velocity

A reduction in muscle fibre conduction velocity (MFCV) indicates reduced membrane excitability, and is attributed to blood flow reduction (Sjogaard et al. 1988; Zwarts and Arendt-Nielsen 1988), reduced pH (Mortimer et al. 1970) and the simultaneous increase of extracellular K^+^ and intracellular Na^+^ (Hodgkin and Katz 1949; Overgaard et al. 1997). Therefore, reports of MFVC and membrane excitability in humans may vary with the use of (a) resting membrane potential (Hodgkin and Horowicz 1959), (b) EMG spectral parameters or (c) M-wave amplitude. Costill et al. (1976) calculated the resting muscle membrane potential and reported no change in membrane excitability after dehydration of ~ 6% of body mass; however, these findings were taken from rested muscle. At warm (~ 37 °C) muscle temperatures, the opening and closing of voltage-gated Na^+^ channels is accelerated, which allows less Na^+^ to enter the cell, leading to a more rapid onset depolarization and faster MFVC (Rutkove et al. 1997). Hypo-hydration (independent of heat) is reported to reduce MFCV (Bigard et al. 2001) as indicated by reductions in EMG mean power frequency (Lindstrom and Magnusson 1977). Conversely, Bowtell et al. (2013) reported an increase in sarcolemma excitability in active muscles after hypo-hydration despite a reduction in HRT and peak force production. This was not observed in the euhydrated group and similar to Costill et al. (1976), was not observed during rest, indicating increased MFCV to be an insufficient driver of force production during a MVC after hypo-hydration. Therefore, it is suggested that independent of heat, hypo-hydration may lead to an increased MFCV, yet despite this, muscle contractility is reduced.

**Fig. 1:**
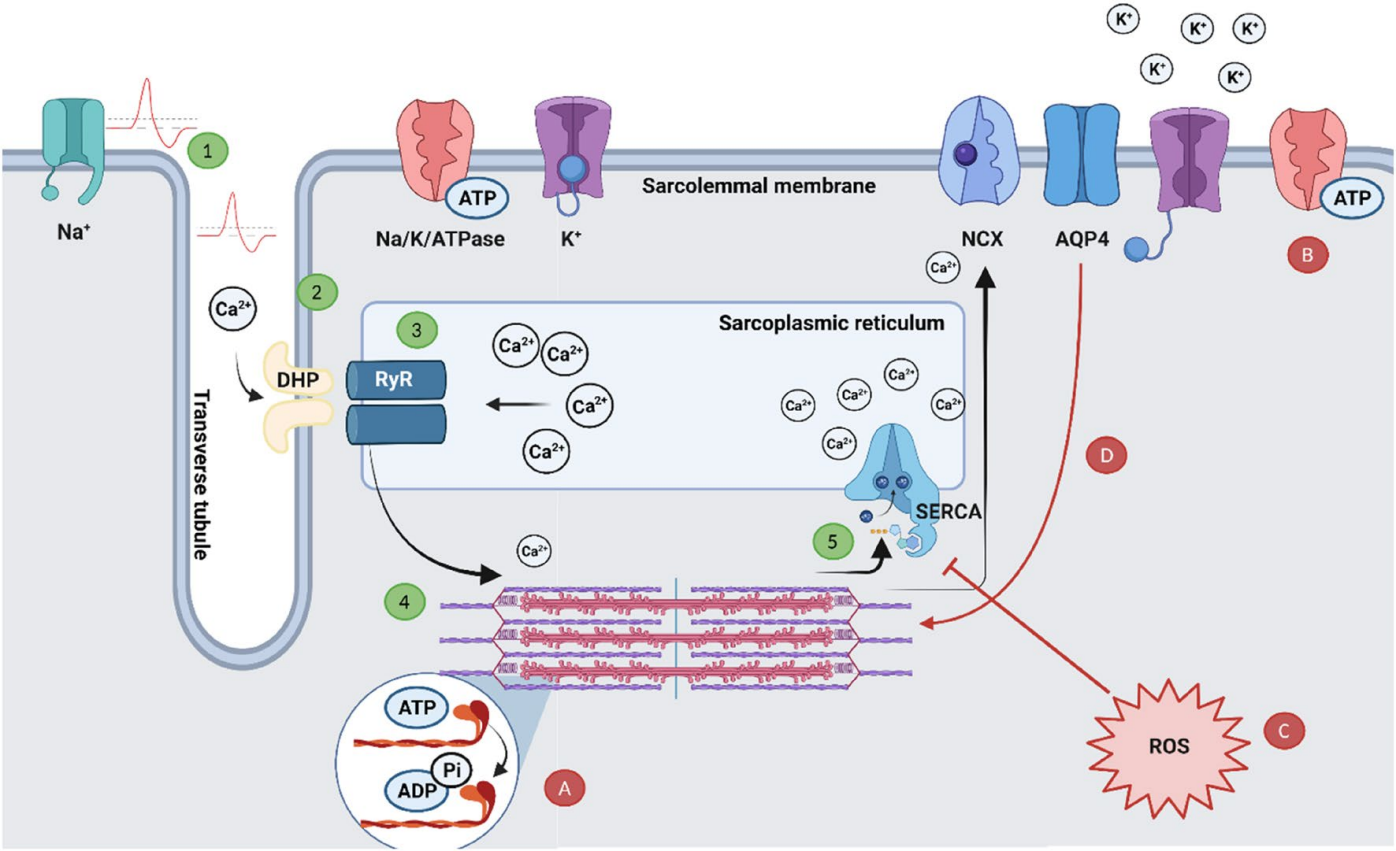
Typical release and re-uptake of Ca^2+^ in the sarcolemma (1–5), and proposed mechanisms of impaired contractility (**A**–**D**). (1) Action potential propagates down the transverse tubule. (2) DHP/LTCC senses membrane depolarization and activates RyR on SR. (3) RyR briefly opens to release a pulse of Ca^2+^. (4) Ca^2+^ bonds to troponin, activating cross-bridge cycle. (5) During relaxation, SERCA pump remove Ca^2+^ from the myofilaments to restore SR Ca^2+^ levels, some may enter into mitochondria or be removed by NCX. **A**, **B** Combination of cellular shrinkage, increased need for ATP hydrolysis at myosin heads and Na^+^/K^+^/ATPase pump, and extracellular K^+^ accumulation might reduce contractility, through impaired cross-bridge cycle function and ability to repolarise and hyperpolarise the cell membrane in time to propagate further action potentials. **C** The increase in ROS from increased blood viscosity and shear stress inhibits SERCA activity, thus reducing Ca^2+^ reuptake into the SR (Lehoux 2006; Powers and Jackson 2008; Connes et al. 2013). **D** A reduction in AQP4 channels may alter lattice spacing of myofilaments and alter protein expression related to Ca^2+^ reuptake (Basco et al. 2011; Farhat et al. 2018). *ADP* adenosine di-phosphate, *AQP4* aquaporin 4, *ATP* adenosine tri-phosphate, *DHP/LTCC* dihydropyridine/L-type calcium channel, *NCX* Na^+^/Ca^2+^ exchanger, *RyR* ryanodine receptor, *SR* sarcoplasmic reticulum, *SERCA* sarcoplasmic/endoplasmic reticulum calcium ATPase, *ROS* reactive oxygen species

A higher MFVC is associated with higher ATP hydrolysis by myofibrillar ATPase at the myosin heads (Gray et al. 2006). In addition, an increase in action potential propagation results in the efflux of extracellular K^+^ (Sjogaard et al. 1985). Therefore, there is an increased demand for ATP hydrolysis for the ECC process, as well facilitating the Na^+^ K^+^ adenosine triphosphatase (Na^+^/K^+^/ATPase) pump, to restore ionic balance. The combination of cellular shrinkage, increased need for ATP hydrolysis and extracellular K^+^ accumulation may explain a reduction in contractility, through a reduced cross-bridge cycle function and ability to repolarise and hyperpolarise the cell membrane in time to propagate further action potentials (Allen et al. 2008). Perhaps, a slower Ca^2+^ reuptake and longer repolarisation times [as indicated through prolonged half relaxation time (Bowtell et al. 2013)], is a result of reduced capacity or slower activation of Na^+^/K^+^/ATPase pumps to defend intracellular water volume (Fig. 1A, B). In summary, maintaining adequate force production after hypo-hydration, may rely on higher ATP hydrolysis, which may be limited as a result of protecting intracellular water.

### Impaired Ca^2+^ reuptake and excitation–contraction-coupling

Since the lengthening of muscle relaxation time is related to reduced Ca^2+^ re-uptake (Gollnick et al. 1991), it is of interest that the production of reactive oxygen species (ROS) inhibits sarco/endoplasmic reticulum ATPase (SERCA) pump activity, subsequently reducing Ca^2+^ reuptake into the SR (Powers and Jackson 2008). Indeed, hyperthermia and hypo-hydration are reported to increase ROS production via various mechanisms, such as increased blood viscosity and endothelial shear stress (van der Poel and Stevenson 2007; Paik et al. 2009; Hillman et al. 2011; Laitano et al. 2012; Georgescu et al. 2017). In addition, sweat losses, fluid shifts and increased blood osmolality leads to a change haemoconcentration and viscosity (Vandewalle et al. 1988), resulting in shear stress along the vascular walls and the subsequent release of nitric oxide (Connes et al. 2013) and ROS (Lehoux 2006). Irrespective of an increase in peripheral and corticospinal excitability, it is hypothesised that the muscle contractile units are unable to utilise the neural drive, owing to reduced intra-cellular Ca^2+^ reuptake (Fig. 1C). This would result in decreased force production and increased muscle HRT (Bowtell et al. 2013) or accelerated fatigue during repeated contractions (Bigard et al. 2001).

An alternative hypothesis related to reduced Ca^2+^ handling consists of specialised water channels in skeletal muscle (aquaporins). Aquaporin-4 (AQP4) is a crucial water channel of the neuromuscular system, particularly found in the sarcolemma of fast twitch fibres and determines muscle permeability (Frigeri et al. 1995, 1998). Farhat et al. (2018) observed more than a 50% decline in AQP4 expression in rodent fast-twitch fibres after 96-h water deprivation. In animals, the absence of AQP4 channels in muscle fibres has been reported to alter protein expression related to Ca^2+^ handing, buffering and glycolytic metabolism (Basco et al. 2011), resulting in impaired voluntary exercise (Basco et al. 2010). In addition, Gulati and Babu (1982) observed a reduction in maximal isometric force after exposing frog muscle fibres to a hypertonic solution; this was associated with reduced fibre width and altered lattice spacing of thick and thin filaments in the sarcolemma. Lattice spacing is a crucial regulator of force generation via the muscle length-tension relationship (Williams et al. 2013). It is hypothesised that AQP4 may determine muscle-specific responsiveness to hyperosmolality, thus reducing cross-sectional area and altering lattice spacing in fast-twitch muscle fibres, subsequently reducing muscle force (Farhat et al. 2018) (Fig. 1D). However, further research is required on human muscle fibres to determine the effects of in vivo hypo-hydration.

### Altered neural drive and contraction-specific fatigue

It is also possible that the effect of hypo-hydration on skeletal muscle is dependent on contraction and/or fibre type, which further rely on glycogen breakdown or a sustained Ca^2+^ re-uptake in the SR (Farhat et al. 2018). Hypo-hydration has not been shown to reduce muscle strength, nor alter phosphocreatine recovery or H^+^ concentration (Montain et al. 1998); though, it could feasibly increase phosphocreatine and muscle glycogen utilisation (Montain et al. 1998; Hargreaves et al. 1996). However, reductions are notably observed during the performance of repeated, strength-endurance protocols (Montain et al. 1998; Bigard et al. 2001; Barley et al. 2018) and high-intensity endurance performance (Judelsen et al. 2007). Approximately 2.7 g of water are bound to 1 g of glycogen (Sherman et al. 1982); therefore, muscle contractions relying on glycogenolysis will facilitate the movement of water molecules from the intra to extracellular space (Olsson and Saltin 1970). A reduction in AQP4 channels could present a challenge for muscle fibres that rely on rapid and efficient water and Ca^2+^ turnover (see “Impaired Ca2+ reuptake and excitation–contraction-coupling” section and Fig. 1), thus reducing force output and time to fatigue during repeated contractions. In addition, hypo-hydration may influence specific contraction types, potentially indicating distinct locations and mechanisms of failure. Hayes and Morse (2010) investigated the dose response of hypo-hydration on muscle performance and reported a reduction in isometric force after one exposure (1% body mass loss), yet isokinetic force was either unchanged or reduced after three exposures or more. It was suggested that concentric contractions at a high velocity may not be as susceptible to hypohydration-induced decrements as slow isokinetic or isometric contractions (Hayes and Morse 2010). Similarly, Bowtell et al. (2013) reported the reduction of peak isometric and eccentric, but not concentric torque (Bowtell et al. 2013). Since eccentric and isometric contractions are less reliant on motor unit activation and energy expenditure (Coburn et al. 2006; Hoppeler 2016; Hody et al. 2019), this indicates performance decrements during brief eccentric contractions to be a result of contractile failure, as opposed to a reduction in central drive or substrate depletion. Therefore, it is likely that hypo-hydration modulates force production according to the type of contraction; a supposition further supported by the selective responsiveness in fast-twitch muscle fibres and alterations to contractile elements (see “Impaired Ca2+ reuptake and excitation–contraction-coupling” section).

The vast majority of studies investigating neuromuscular function utilise isometric contractions, therefore it is important to note that isometric exercise involves the occlusion of blood flow to active muscle, depending on the intensity of contraction (Barcroft and Millen 1939; Edwards et al. 1972). The metabolic and resultant ischemic environment increases local muscle temperature and stimulates chemo- and mechanoreceptor activity (Barnes 1980; Sejersted et al. 1984), resulting in afferent stimulation of sympathetic nervous activity (Seals and Victor 1991). The combination is thought to depress motor unit firing rates (Garland and McComas 1990; Woods et al. 1987), thereby modifying the relationship between central neural drive and motor unit recruitment (Bigland-Ritchie et al. 1986, Woods et al. 1987). Motor unit discharge rates are proportionate to the synaptic input they receive (Enoka and Duchateau 2017), but in addition to ionotropic input, rate coding may be influenced by neuromodulatory input (e.g., noradrenaline) to the motor neuron pool via persistent inward currents (Heckman and Enoka 2012; Perrier and Cotel 2015; Aston-Jones and Waterhouse 2016). However, noradrenaline has not been associated with changes in sarcolemma excitability nor motor neuron discharge activity (Plewnia et al. 2001, 2002; Ilić et al. 2003; Boroojerdi et al. 2001; Strahlendorf et al. 1980; Fung and Barnes 1981). Therefore, an alternative theory related to the reduction in force despite reduced cortical inhibition and unaltered corticospinal excitability after hypo-hydration (Bowtell et al. 2013), is attributed to the increase in sympathetic nerve activity (to preserve vasomotor function; Buharin et al. 2013). In summary, sympathetic nerve activity could result in altered neural drive (i.e., reduced cortical inhibition or unaltered corticospinal excitability) as observed after hypo-hydration (Bowtell et al. 2013), yet has no effect on muscle function.

During an MVC, the reported effects of hypo-hydration are extremely varied (see “Muscle contractility-specific responses” section), however, when analysing the specific role of the CNS and PNS, some have reported a lower cSP, increased sarcolemma excitability (Bowtell et al. 2013), unchanged (Periard et al. 2012; Barley et al. 2018) or increased central motor drive (Bigard et al. 2001), and higher mean power frequency (Vallier et al. 2005) relative to euhydrated controls. Despite this, force reductions continue to persist. Interestingly, Periard et al. (2012) and Barley et al. (2018) reported a decline in force production during repeated MVCs, not associated with VA, indicating a loss of force to be unrelated to voluntary central drive and more likely to be a result of alterations to the peripheral musculature. Therefore, an alternative view of heat-induced hypo-hydration is proposed as: (a) central drive may be enhanced via reduced cortical inhibition or increased cortical facilitation, in an attempt to compensate for potential force decrements when hypo-hydrated but, (b) this may not be sufficient, particularly during sustained and repeated voluntary contractions where contractile function is impaired (Todd et al. 2005). This may explain why heat-induced hypo-hydration is notably reported to have ‘no effect’ on brief measures of power and strength (Jacobs 1980; Hoffman et al. 1995; Cheuvront et al. 2006; Watson et al. 2005; Periard et al. 2012; Greiwe et al. 1998; Montain et al. 1998; Evetovich et al. 2002), but consistently impairs performance during repeated or sustained contractions (Bigard et al. 2001; Maxwell et al. 1999; Mohr et al. 2010; Judelsen et al. 2007; Kraft et al. 2010; Periard et al. 2012; Bosco et al. 1968; Torranin et al. 1979; Schofstall et al. 2001). Further studies are required to elucidate the facilitatory and inhibitory responses (in the corticospinal pathway) to hypo-hydration.

### Supraspinal fatigue, increased perception of effort and activation of pain-related networks

Supraspinal fatigue is defined as loss of force caused by suboptimal output from the motor cortex (Taylor et al. 2006). Hypo-hydration also notably increases perceptions of fatigue, tension, and anxiety (Ganio et al. 2011; Sharma et al. 1986; Gopinathan et al. 1988; Tomporowski et al. 2007). Conscious signals originating from both central and peripheral afferent pathways could mediate behaviour and reduce motivation to minimize discomfort (Cabanac 2006). Heat-induced hypo-hydration resulting in a 4% body weight loss resulted in no change of muscle strength, despite a 15% reduction in time to fatigue. Interestingly, hypo-hydration did not exacerbate muscle pH, hydrogen ion and inorganic phosphate accumulation during the fatiguing task, thus it was proposed that hypo-hydration may result in an inability or unwillingness to sustain force production, despite adequate muscle strength (Montain et al. 1998). Furthermore, the negative psychological associations attributable to thirst may act as a signalling mechanism to promote a greater conscious perception of effort thus, invoking a behavioural change to reduce physical effort (Edwards et al. 2007). Alternatively, force may be maintained but only at the expenditure of higher metabolic cost, as seen in increased blood-oxygen-dependant-level activation (using fMRI) of the fronto-parietal brain region during a cognitive task (Kempton et al. 2011). Therefore, it is suggested that hypo-hydration may negatively affect motivation and increase effort perception, resulting in reduced central motor drive during exercise.

**Fig. 2:**
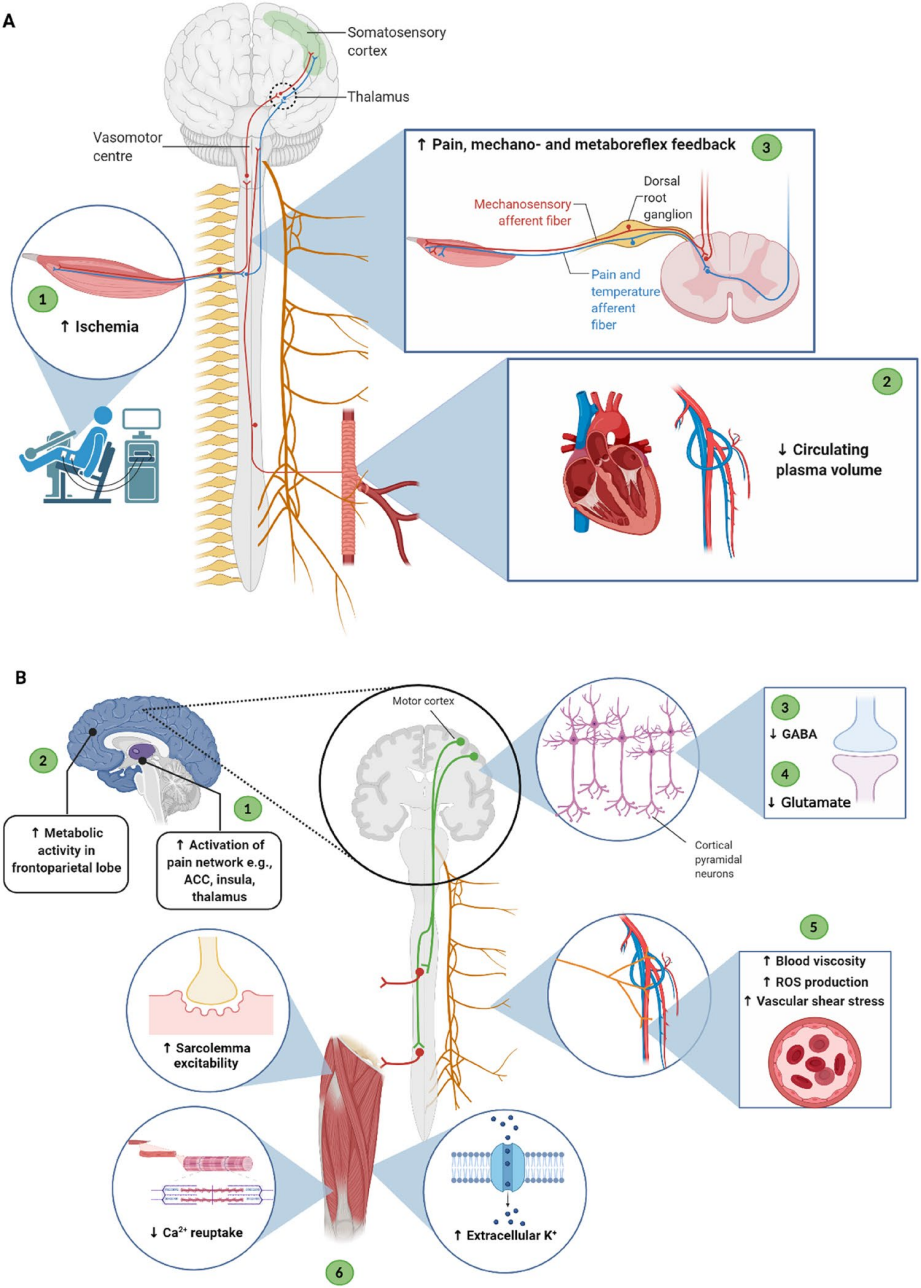
Summary of proposed afferent (**A**) and efferent (**B**) responses to heat-induced hypo-hydration during an MVC. **A** Afferent responses: (1) ischemia as a result of increased/prolonged contractions. (2) Reduced plasma volume due to water losses trigger a vasomotor response. (3) Upon an MVC, there is an increase in pain, mechano- and metaboreflex feedback sent to the thalamus and somatosensory cortex to alter behaviour and central motor drive. **B** Efferent responses: (1) increased activation of pain network due to reduced pain threshold (Ogino et al. 2014). (2) Increased metabolic activity in other brain regions (e.g., frontoparietal lobe) due to increased effort perception (Kempton et al. 2011). (3) Reduced GABA to compensate for force losses in contractile units (Bowtell et al. 2013). (4) Reduced glutamate and central motor drive in conscious reduction of effort (loss in motivation or increased pain) (St Clair Gibson et al. 2013). (5) Volume changes result in increased blood viscosity, vascular shear stress and ROS production (Van der Poel and Stevenson 2007; Hillman et al. 2011; Laitano et al. 2012; Paik et al. 2009; Vandewalle et al. 1988; Connes et al. 2013; Lehoux, 2006). (6) Impaired contractile function (contraction-dependent) due to increased need for ATP hydrolysis and reduced Ca^2+^ reuptake in SR (see Fig. 1). *GABA* *γ*-aminobutyric acid, *ROS* reactive oxygen species

Hypo-hydration has also been shown to enhance activation of pain-related brain networks (Ogino et al. 2014) and increase pain perception (Moyen et al. 2015; Perry et al. 2016; Bear et al. 2016). The cold pressor test is commonly used to assess autonomic outflow to the extremities (Victor et al. 1987) and involves the immersion of a limb in cold water, thus inducing high levels of pain (Di Piero et al. 1994; Zvan et al. 1998). Perry et al. (2016) reported a modified cerebrovascular response to the cold pressor test in hypo-hydrated subjects due to increased pain perception. Furthermore, Ogino et al. (2014) observed the effects of a 12-h fasting and 40-min exercise protocol, resulting in increased activation of the anterior cingulate cortex, insula, and thalamus, alongside increased thirst, and reduced pain threshold during the cold pressor test. Interestingly, Farrell et al. (2006) found similar brain areas were activated after inducing pain and thirst via noxious pressure and infused hypertonic saline, respectively, but activation of the pregenual cingulate and orbitofrontal cortices occurred in the combined presence of thirst and pain, suggesting an integrative role of thirst and pain sensation. Minor discomfort is also sensed at the onset of a contraction, developing into severe discomfort and pain over time that alters the perception of sensations in the contracting musculature (Bigland-Ritchie et al. 1978). Experimentally induced pain (EIP) via intramuscular injections of hypertonic saline, is proposed to invoke similar nociceptive pathways of exercise-induced pain (Laursen et al. 1999; O’Connor and Cook 1999). Current evidence suggests EIPs to reduce muscle strength (Graven-Nielsen and Arendt-Nielsen 2008; Henriksen et al. 2011; Stackhouse et al. 2013) and submaximal force steadiness (Graven-Nielsen et al. 1997; Rice et al. 2015) indicating increased nociceptive activity to be a cause of force decrements. Interestingly, Graven-Nielsen et al. (2002) demonstrated that EIP reduced maximal voluntary torque, despite an unaffected twitch torque, implying that performance decrements were due to mechanisms residing in the CNS rather than the peripheral musculature (Graven-Nielsen et al. 2002). Indeed, EIP is shown to modify corticospinal and intracortical excitability (Le Pera et al. 2001; Schabrun and Hodges 2012), emphasising the strong relationship between the nociceptive and motor systems, however, the relationship with hypo-hydration is yet to be explored. A summary of all the proposed mechanisms can be found in Fig. 2.

## Future directions

A major limitation to understanding the effects of hypo-hydration on neuromuscular function is the method of inducing fluid loss. Typically, hypo-hydration is achieved using active (exercise) or passive protocols in temperate conditions, thus resulting in an elevated core temperature and exercise-induced fatigue. Such protocols represent ecologically valid scenarios of exercise under heat-stress, e.g., running/cycling in temperate conditions or methods of rapid weight loss in combat sports, however, it is difficult to isolate the effects of hypo-hydration. In addition, a methodological limitation of many heat-induced hypo-hydration studies, is (a) to not report the return of core temperature to baseline and (b) not observe the effects of fluid restoration thereafter; this results in a lack of consistency across findings attributed to hypo-hydration. Furthermore, studies utilising diuretics (e.g., furosemide) result in hypo-hydration (iso-osmotic hypovolemia) dissimilar to heat-induced hypo-hydration (hyperosmotic hypovolemia), meaning that the mechanisms of performance impairment are unlikely to be the same. Consequently, future studies should differentiate the effects of hypo-hydration from hyperthermia and exercise-induced fatigue, similar to the methods of Periard et al. (2012) and van den Heuvel et al. (2020). Furthermore, future studies should investigate the brain’s intracortical inhibitory and excitatory activity (via paired-pulse TMS) and motor unit activity (via high-density surface EMG) to elucidate the distinct roles of the central and peripheral nervous systems during force output, following heat-induced hypo-hydration.

## Conclusion

The present evidence suggests that heat-induced hypo-hydration leads to a notable reduction in neuromuscular function, particularly during repeated and sustained contractions. Moreover, hypo-hydration may lead to altered corticospinal excitability (via reduced corticospinal inhibition), which might act as a compensatory mechanism to minimise force loss during an MVC, but this is insufficient during repeated contractions due to failure at the contractile level. This review has provided an overview of the neurophysiological responses to heat-induced hypo-hydration, its effects on neuromuscular function and the potential underlying mechanisms.

## Abbreviations

AQP4: Aquaporin-4
ATP: Adenosine tri-phosphate
CNS: Central nervous system
CSE: Corticospinal excitability
cSP: Cortical silent period
ECC: Excitation–contraction coupling
EEG: Electroencephalography
EMG: Electromyography
GABA: γ-Aminobutyric acid
fMRI: Functional magnetic resonance imaging
HRT: Half-relaxation time
MFVC: Muscle fibre conduction velocity
Mmax: Maximum motor unit potential
MNS: Motor nerve stimulation
MVC: Maximum voluntary contraction
PNS: Peripheral nervous system
ROS: Reactive oxygen species
RWL: Rapid weight loss
SERCA: Sarco/endoplasmic reticulum ATPase
VA: Voluntary activation
VA_MNS_: Voluntary activation measured using motor nerve stimulation
VA_TMS_: Voluntary activation measured using motor cortex stimulation
TMS: Transcranial magnetic stimulation
